# *In Vitro* Evaluation of the Risk of Inducing Bacterial Resistance to Disinfection Treatment with Photolysis of Hydrogen Peroxide

**DOI:** 10.1371/journal.pone.0081316

**Published:** 2013-11-25

**Authors:** Hiroyo Ikai, Yu Odashima, Taro Kanno, Keisuke Nakamura, Midori Shirato, Keiichi Sasaki, Yoshimi Niwano

**Affiliations:** Tohoku University Graduate School of Dentistry, Aoba-ku, Sendai, Japan; University Paris South, France

## Abstract

The purpose of the present study was to evaluate the risk of inducing bacterial resistance to disinfection treatment with photolysis of H_2_O_2_ and comparing this with existing antibacterial agents. We tested seven antibacterial agents, including amoxicillin, cefepime hydrochloride, erythromycin, ofloxacin, clindamycin hydrochloride, ciprofloxacin hydrochloride, and minocycline hydrochloride, as positive controls for validation of the assay protocol. For all of the agents tested, at least one of the four bacterial species (*Staphylococcus aureus*, *Enterococcus faecalis*, *Escherichia coli*, and *Streptococcus salivarius*) was resistant to these agents by repeated exposure to subinhibitory concentrations of the agents up to 10 times. In contrast, antibacterial activity against any of the bacterial species tested (*S. aureus*, *E. faecalis*, *E. coli*, *S. salivarius*, *Pseudomonas aeruginosa*, *Streptococcus mutans*, and *Aggregatibacter actinomycetemcomitans*) was not affected by repeated exposure to the disinfection treatment up to 40 times. This finding suggested that the risk of inducing bacterial resistance by disinfection treatment was low. The active ingredient of this disinfection treatment is hydroxyl radicals generated by photolysis of H_2_O_2_. Therefore, hydroxyl radicals interact with several cell structures and different metabolic pathways in microbial cells, probably resulting in a lack of development of bacterial resistance. In conclusion, disinfection treatment with photolysis of H_2_O_2_ appears to be a potential alternative for existing antimicrobial agents in terms of a low risk of inducing bacterial resistance.

## Introduction

Disinfection treatment, in which hydroxyl radicals generated by photolysis of hydrogen peroxide (H_2_O_2_) kill bacteria efficiently, has been developed in our laboratory [1], [2]. *In vitro* studies found that *Staphylococcus aureus*, *Streptococcus mutans*, *Enterococcus faecalis*, and *Aggregatibacter actinomycetemcomitans* were killed with a >5-log reduction of viable counts within 3 min when bacterial suspension in 1 M H_2_O_2_ was irradiated with laser light at 405 nm [1]. One molar H_2_O_2_ corresponds to approximately 3%, which is a concentration used as a disinfectant for skin and oral mucosa. A subcommittee of the US Food and Drug Administration also concluded that H_2_O_2_ is safe at concentrations of up to 3% [3]. In addition to *in vitro* findings, an *in vivo* antibacterial effect of this disinfection system was proven effective in a rat model of superficial *S. aureus* infection [4].

Antibiotic-resistant bacteria are continuously emerging because of the widespread and sometimes indiscriminate use of antibiotics in the medical field [5], [6]. Reactive oxygen species (ROS), such as hydroxyl radicals and singlet oxygen, non-specifically oxidize several cell structures, leading to cell death [7]–[9]. Consequently, it is unlikely that bacteria would develop resistance to the cytotoxic action of ROS [7]–[10]. Therefore, disinfection treatment using photolysis of H_2_O_2_ is not expected to induce bacterial resistance to this treatment either.

To assess the risk of developing bacterial resistance to antibiotics and antiseptics, monitoring minimal inhibitory concentrations (MICs) of these agents after serial passage of culture through subinhibitory concentrations of these agents has proven effective [11]–[13]. Therefore, in the present study, clinically available antibacterial agents were used as positive controls to validate the assay protocol. This was performed by evaluating if the test bacterial strains used in the present study would develop resistance to the agents by repeated exposure to subinhibitory concentrations of the agents.

The purpose of the present study was to determine if the risk of developing bacteria resistant to disinfection treatment using photolysis of H_2_O_2_ is low through repeated exposure of bacteria under the sublethal conditions in which the bacteria were not completely killed.

## Materials and Methods

### Bacteria

*S. aureus* JCM 2413, *E. faecalis* JCM 7783, *Escherichia coli* JCM 5491, *Streptococcus salivarius* JCM 5707, *Pseudomonas aeruginosa* JCM 6119, *S. mutans* JCM 5705, and *A. actinomycetemcomitans* JCM 2434, purchased from the Japan Collection of Microorganisms, RIKEN BioResource Center (Wako, Japan), were used. Suspensions of facultative anaerobic bacteria were prepared from cultures grown on brain heart infusion (BHI) agar (Becton Dickinson Labware, Franklin Lakes, NJ, USA) for *S. aureus*, *E. faecalis*, *E. coli*, and *S. salivarius*, and on desoxycholate-hydrogen sulfide-lactose (DHL) agar (Nissui, Tokyo, Japan) for *P. aeruginosa* aerobically at 37°C for 20 h. Suspensions of *S. mutans* and *A. actinomycetemcomitans* were from cultures grown anaerobically on BHI agar using the Anaero Pack (Mitsubishi Gas Chemical Company, Tokyo, Japan) at 37°C for 44 h. The viable count of each bacterial suspension in each antibacterial assay was adjusted to a given density as described in the following sections using a colorimeter (WPA CO7500 colorimeter, Biochrom, Cambridge, UK).

Susceptibility testing for antibacterial agents and repeated exposure of bacteria to the agents.

**Figure 1:**
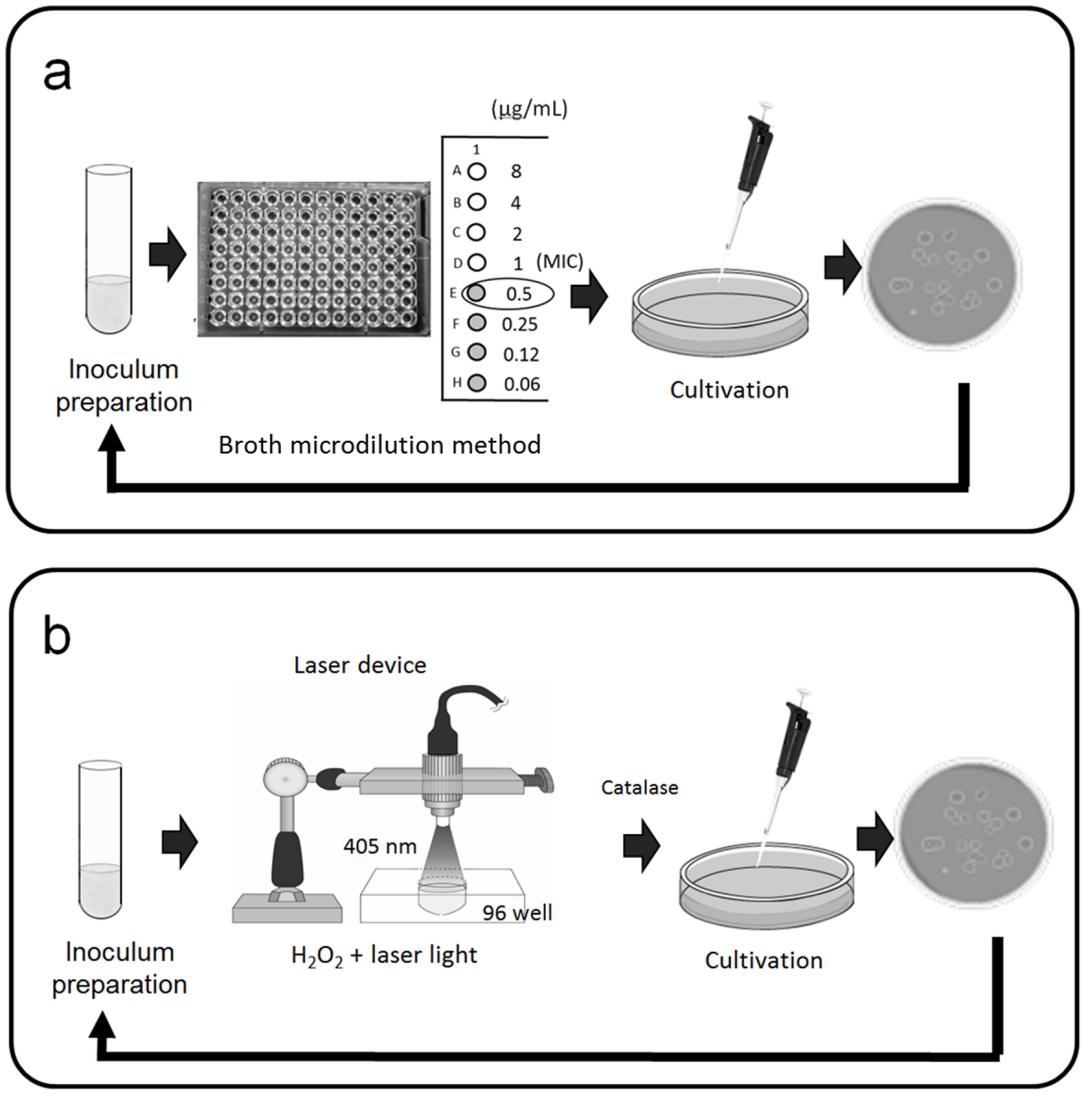
Schematic illustrations of susceptibility testing. (a) Antibacterial agents and (b) disinfection treatment with photolysis of H_2_O_2_ are shown.

Microdilution plates in which antibacterial agents were dehydrated were custom fabricated by Eiken Chemical Co., Ltd. (Dry Plate Eiken, Tokyo, Japan) for a broth microdilution method to determine MICs as described by the Clinical and Laboratory Standards Institute M7-A7 [14]. The following seven antibacterial agents provided by Eiken Chemical Co., Ltd. were tested: a β-lactam antibiotic, amoxicillin (AMX), a cephem antibiotic, cefepime hydrochloride (CFPM), a macrolide antibiotic, erythromycin (EM), a fluoroquinolone antibiotic, ofloxacin (OFLX), a lincosamide antibiotic, clindamycin hydrochloride (CLDM), a fluoroquinolone antibiotic, ciprofloxacin hydrochloride (CPFX), and a tetracycline antibiotic, minocycline hydrochloride (MINO). Figure 1a shows a schematic illustration of the assay method. In brief, each bacterial species (*S. aureus*, *E. faecalis*, *E. coli*, and *S. salivarius*) grown on BHI agar was harvested and suspended in Muller-Hinton broth (Kanto Chemical Co., Inc., Tokyo, Japan). The number of colony-forming units (CFU) of each strain was adjusted to 1×10^5^ CFU/mL. An aliquot (100 µL) of the resultant suspension was inoculated into a well of the plates. After incubation with a lid at 37°C for 20 h, bacterial growth was visually assessed to determine the MIC using a microplate reading mirror (Eiken Chemical Co., Ltd.). After determining the initial MICs, 20 µL of a bacterial suspension of a well showing 1/2 MIC was mixed with 1980 µL of Muller-Hinton broth to eliminate the effect of drug carry-over. A volume of 20 µL of the resultant suspension was then inoculated onto BHI agar followed by incubation at 37°C for 20 h. Bacterial suspensions were again prepared and MICs were determined as described above. The same procedure was repeatedly performed to assess the induction of bacterial resistance to the antibacterial agents tested (total number of treatments = 10). In the case of inconvenience for continuous working, a mixture of 20 µL of a bacterial suspension of a well showing 1/2 MIC and 1980 µL of Muller-Hinton broth was kept at 4°C until the next assay. An increase of four times or higher in MIC over the initial MIC was set as the criterion for inducing resistance to each antibacterial agent [15]. All tests were performed in duplicate (two independent assays).

### Susceptibility testing for disinfection treatment with photolysis of H_2_O_2_ and repeated exposure of bacteria to this treatment

Disinfection treatment with photolysis of H_2_O_2_ was performed according to our previous study [1]. A continuous-wave laser device (RV-1000; Ricoh Optical Industries, Hanamaki, Japan) was used to photolyze H_2_O_2_. Three percent (w/v) H_2_O_2_ was prepared by diluting 31% (w/v) H_2_O_2_ (Santoku Chemical Industries, Tokyo, Japan) with phosphate-buffered saline (PBS, pH 7.4). Bacterial suspensions were prepared in PBS following incubation on the corresponding agar plates as described above, and the initial inoculum size of every bacterial species was adjusted to a range of 5×10^6^ to 1×10^8^ CFU/mL. Figure 1b shows a schematic illustration of the assay method. In a microplate well, 10 µL of the bacterial suspension was mixed with 190 µL of 3% H_2_O_2_ followed by laser light irradiation at 405 nm for 10 to 120 s at an irradiance of 930 mW/cm^2^. Laser light irradiation time was preliminarily determined to obtain an approximately 2-log reduction in viable cell count in each bacterial species. This irradiation time was 120 s for *E. faecalis* and *S. salivarius*, 90 s for *S. aureus* and *S. mutans*, 30 s for *E. coli* and *A. actinomycetemcomitans*, and 10 s for *P. aeruginosa*. We confirmed that exposure of 3% H_2_O_2_ alone (without laser irradiation) for the given time as described above did not exert any bactericidal effect on any of the bacterial species tested. After irradiation, 50 µL of the treated bacterial suspension was added to 50 µL of sterile catalase solution (5000 U/mL) to terminate the bactericidal effect of the remaining H_2_O_2_. A 10-fold serial dilution of the mixture was then prepared using PBS, and 10 µL of the diluted solution was plated on the corresponding agar plate. Agar plates were incubated as described above at 37°C for 20 h or longer to determine the number of CFU/mL. The colonies grown on the agar plates were again suspended in PBS with the inoculum size in the range of 5×10^6^ to 1×10^8^ CFU/mL. The same procedure was then repeatedly performed to assess the induction of bacterial resistance to the treatment (the total number of treatments = 40). All tests were performed in triplicate (three independent assays).

### Electron spin resonance (ESR) analysis for hydroxyl radicals generated by photolysis of H_2_O_2_

To confirm that hydroxyl radicals were generated time-dependently by photolysis of H_2_O_2_, hydroxyl radicals were quantitatively analyzed by an ESR-spin trapping technique as described in our previous studies [1], [16]. In brief, H_2_O_2_ was mixed with 5,5-dimethyl-1-pyrroline *N*-oxide (DMPO; Labotec, Tokyo, Japan), a spin trap agent, in a microplate well to reach final concentrations of 3% (w/v) for H_2_O_2_ and 300 mM for DMPO. The sample was then irradiated with a laser light for 0, 10, 20, and 30 s. After irradiation, the sample was transferred to a quartz cell for ESR spectrometry, and the ESR spectrum was recorded on an X-band ESR spectrometer (JES-FA-100; JEOL, Tokyo, Japan). The measurement conditions for ESR were as follows: field sweep, 331.41–341.41 mT; field modulation frequency, 100 kHz; field modulation width, 0.1 mT; amplitude, 80; sweep time, 2 min; time constant, 0.03 s; microwave frequency, 9.420 GHz; and microwave power, 4 mW. The compound 4-hydroxy-2,2,6,6-tetramethylpiperidine (20 µM; Sigma Aldrich, St. Louis, MO, USA) was used as a standard to calculate the concentration of DMPO-OH, a spin adduct of hydroxyl radicals. The concentration of DMPO-OH was determined using Digital Data Processing (JEOL). All assays were performed in triplicate (three independent assays).

## Results

### Susceptibility testing for antibacterial agents

Table 1 summarizes the MICs at the first, fifth, and tenth exposure of each bacterial species to antibacterial agents tested. The initial MICs of all the seven antibacterial agents against *S. aureus* were within a narrow range between 0.12 and 0.5 µg/mL, and the values become higher at the fifth and tenth exposure. Especially the MICs of CFPN and CLDM at the tenth exposure were 128 and 32 µg/mL, respectively. The initial MICs of the agents against *E. faecalis* and *E. coli* were within a rather wide range (0.5 to 16 µg/mL against *E. faecalis*, and 0.015 to 128 µg/mL against *E. coli*). Prominent increases in MIC were observed in CFPN against *E. faecalis* (from 8 µg/mL at the initial to 128 µg/mL at the tenth) and MINO against *E. coli* (from 0.5 µg/mL at the initial to 16 µg/mL at the tenth). Regarding MICs against *S. salivarius*, MICs of CFPN and MINO could not be obtained because no visible bacterial growth was observed even at the lowest concentration of each agent. Of the seven antibacterial agents, only the MIC of AMX showed 4 times increase during the experiment.

**Table 1:** MICs on the first, fifth, and tenth exposure of each bacterial species to antibacterial agents.

Staphylococcus aureus					Enterococcus faecalis			
Drug	MIC (µg/mL)				Drug	MIC (µg/mL)		
	Initial	5th	10th			Initial	5th	10th
AMX*	0.12	1	2		AMX*	0.5	1	2
CFPN*	0.5	128	128		CFPN*	8	128	128
EM*	0.5	4	8		EM*	2	4	8
OFLX*	0.5	4	4		**OFLX**	2	4	4
CLDM*	0.25	16	32		**CLDM**	16	16	32
CPFX*	0.5	2	2		**CPFX**	1	2	2
MINO*	0.25	2	8		MINO*	2	8	16

Escherichia coli					Streptococcus salivarius			
Drug	MIC (µg/mL)				Drug	MIC (µg/mL)		
	Initial	5th	10th			Initial	5th	10th
**AMX**	4	8	8		AMX*	0.03	0.03	0.12
**CFPN**	1	2	2		**CFPN**	<0.015	<0.015	<0.015
**EM**	32	64	64		**EM**	0.06	0.06	0.12
OFLX*	0.06	0.12	0.48		**OFLX**	2	4	4
**CLDM**	128	128	128		**CLDM**	0.06	<0.015	<0.015
**CPFX**	0.015	<0.015	<0.015		**CPFX**	1	2	2
MINO*	0.5	4	16		**MINO**	<0.12	<0.12	<0.12

Each value represents the mean of duplicate determinations.

Asterisks indicate induction of bacterial resistance to corresponding antibacterial agents as defined by an increase of four times or more in MIC over the initial MIC.

**Figure 2:**
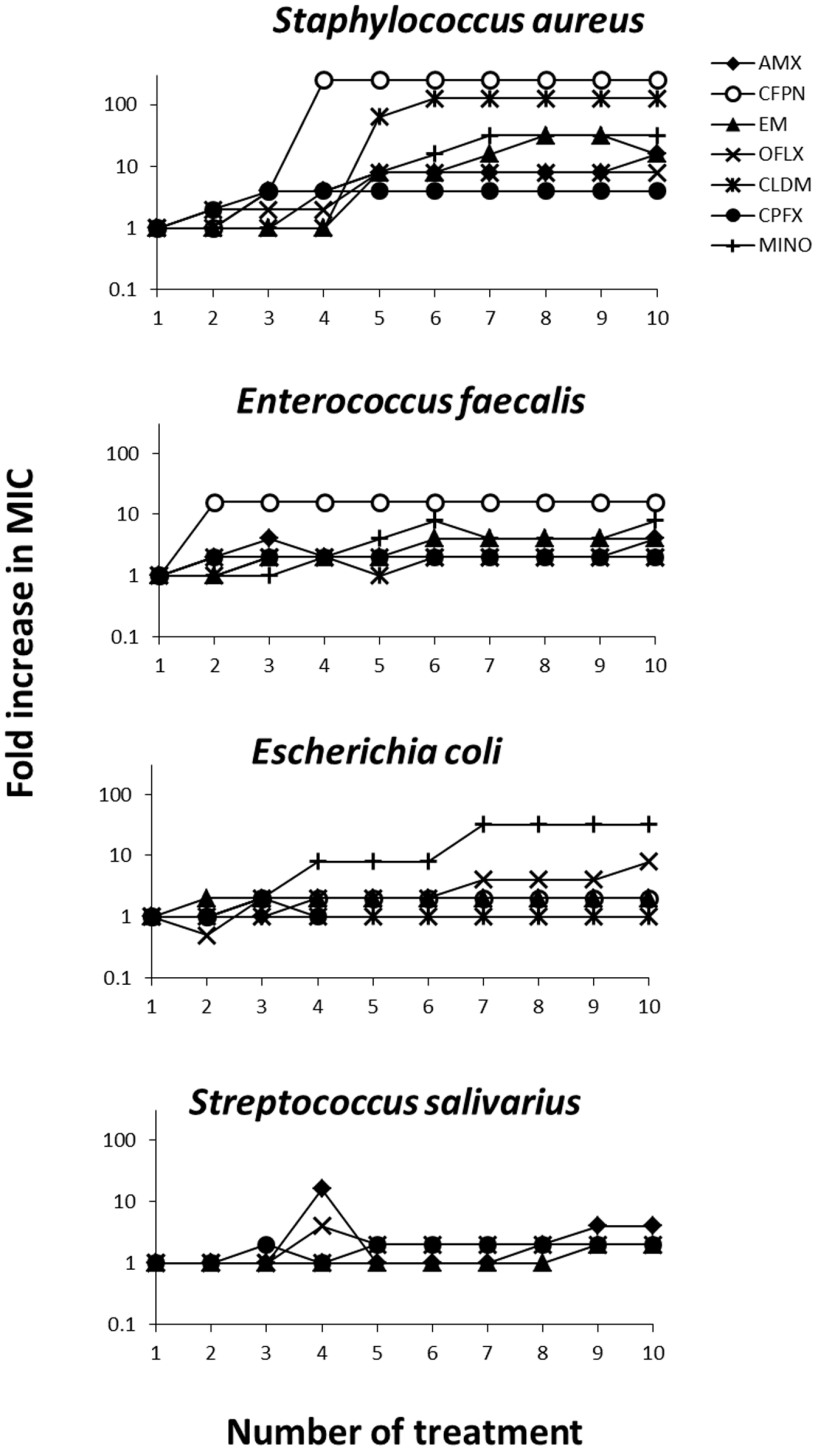
Fold increases in MICs of antibacterial agents against four bacterial species during exposure to these agents. Each bacterial species was exposed 10 times. Each initial MIC is regarded as 1 MIC. Each value represents the mean of duplicate determinations.

To figure out the entire spectrum of inducing bacterial resistance, Fig. 2 shows the changes in the fold increase in MIC in which each initial MIC is regarded as 1 MIC. Of the four bacterial species tested, susceptibility of *S. aureus* prominently lowered with the number of treatment, and all the seven antibacterial agents induced 4- or higher fold increases in MIC. Susceptibility of *E. faecalis* and *E. coli* also lowered in some of the antibacterial agents with the number of treatment. AMX, CFPN, EM, and MINO induced 4- or higher fold increases in MIC against *E. faecalis*, and OFLX and MINO against *E. coli*. Susceptibility of *S. salivarius* tended to be rather stable as compared to that of the other bacterial strains during the experiment.

### Susceptibility testing for disinfection treatment with photolysis of H_2_O_2_

**Figure 3:**
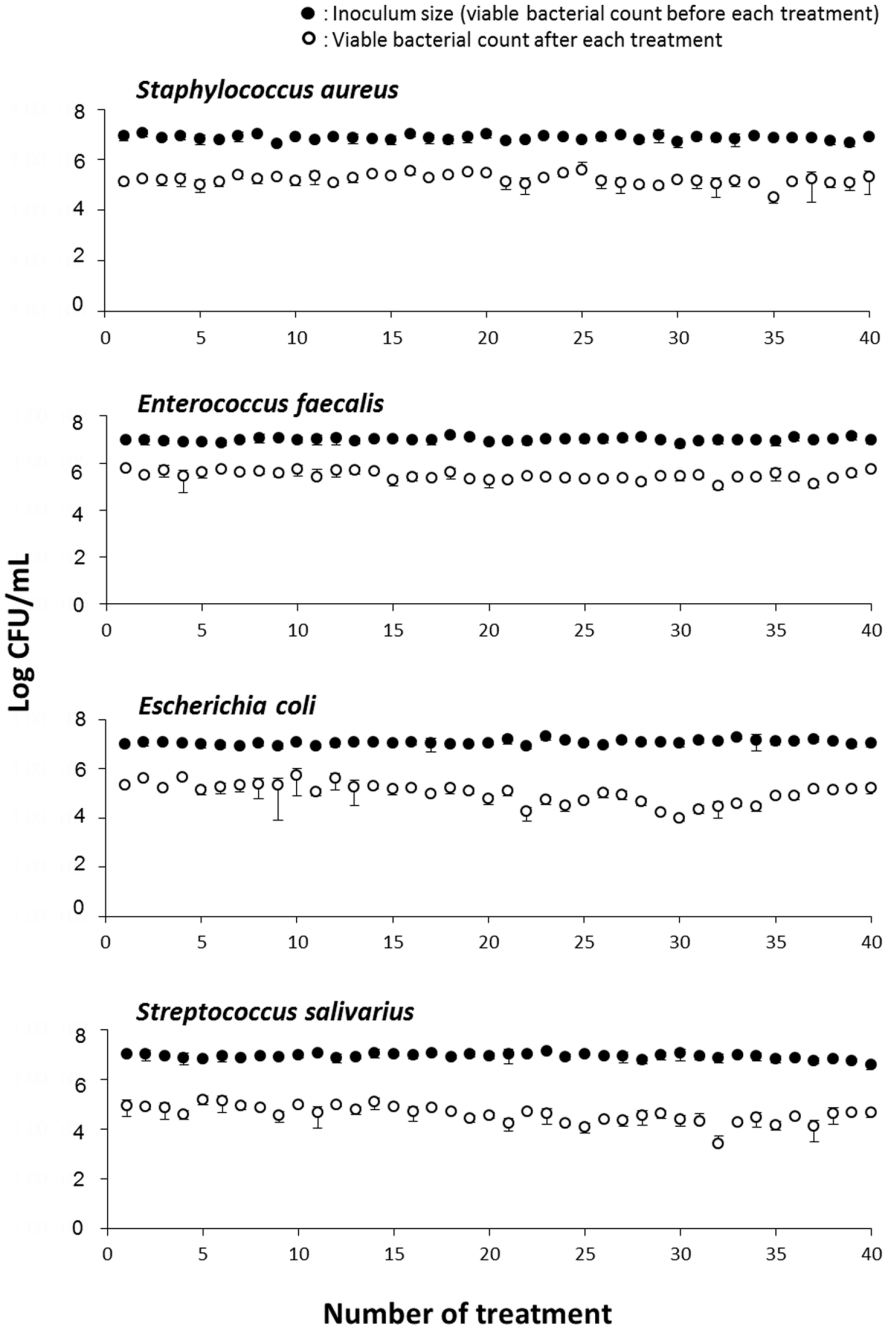
Changes in the antibacterial effect of disinfection treatment with photolysis of H_2_O_2_ in four bacteria. *Staphylococcus aureus*, *Enterococcus faecalis*, *Escherichia coli*, and *Streptococcus salivarius* were exposed 40 times to disinfection treatment. Each value represents the mean ± standard deviation (n = 3).

**Figure 4:**
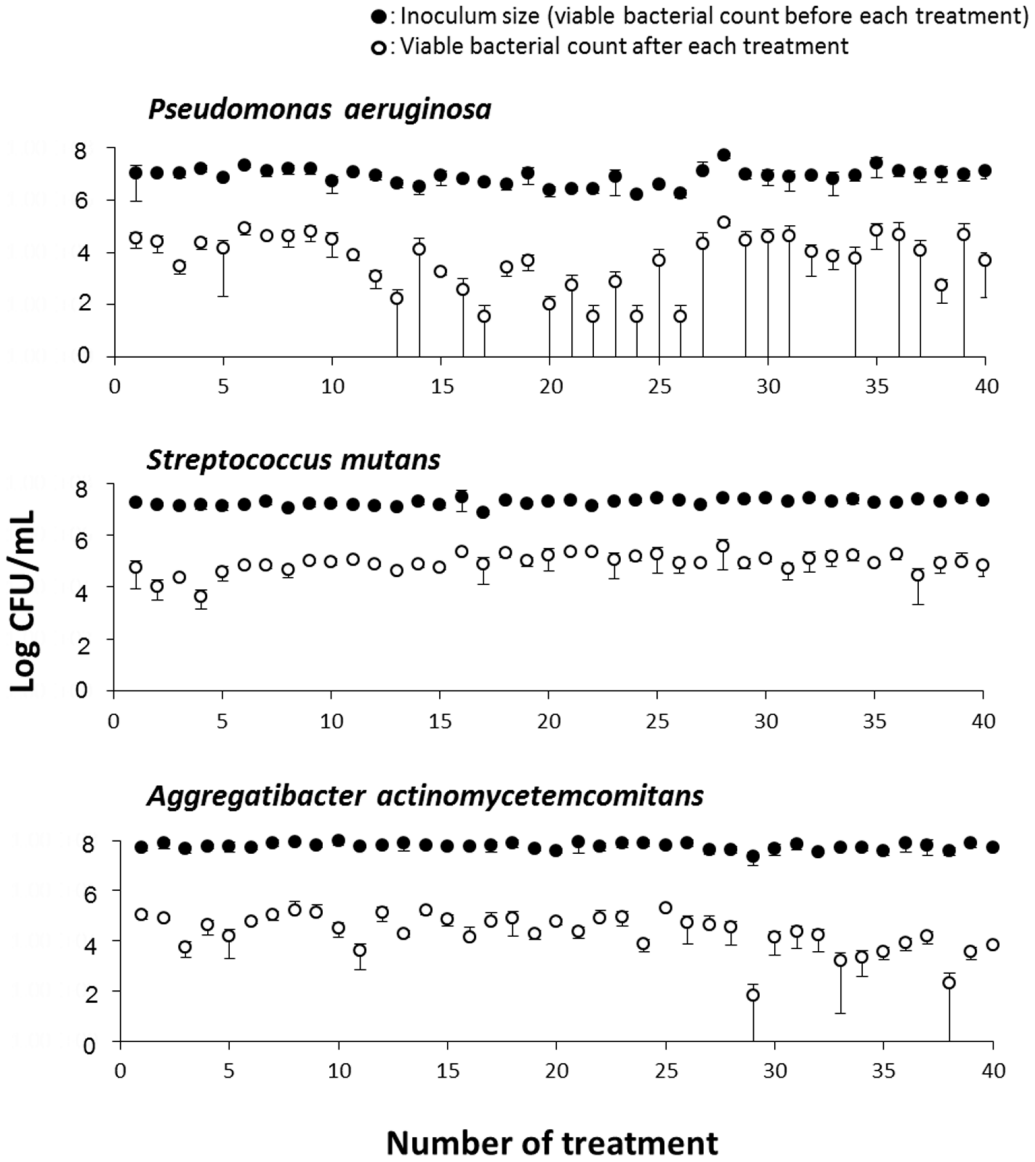
Changes in the antibacterial effect of disinfection treatment with photolysis of H_2_O_2_ in three bacteria. *Pseudomonas aeruginosa*, *Streptococcus mutans*, and *Aggregatibacter actinomycetemcomitans* were exposed 40 times to disinfection treatment. Each value represents the mean ± standard deviation (n = 3).

Figure 3 shows the changes in the antibacterial effect of repeated disinfection treatment with photolysis of H_2_O_2_ in four bacterial species, *S. aureus*, *E. faecalis*, *E. coli*, and *S. salivarius*. When each bacterial species was exposed to the first treatment with photolysis of H_2_O_2_, an approximate 2-log reduction in viable counts was observed. Repeated exposure of bacteria to the treatment of photolysis of H_2_O_2_ did not affect bacterial susceptibility. In addition, the magnitude of the reduction in viable counts in any of the bacterial species tested was mostly within the range of 2- to 3-log order during repeated treatment up to 40 times. Figure 4 shows the changes in the antibacterial effect of repeated disinfection treatment with photolysis of H_2_O_2_ in the three bacterial species, *P. aeruginosa*, *S. mutans*, and *A. actinomycetemcomitans*. Similar to the other four bacterial species described above, an approximate 2-log reduction in viable counts was observed at the first exposure of each bacterial species to treatment with photolysis of H_2_O_2_. Of the three bacterial species, *P. aeruginosa* and *A. actinomycetemcomitans* showed a relatively high susceptibility to this treatment because a laser light irradiation time as short as 10 s for *P. aeruginosa* and 30 s for *A. actinomycetemcomitans*, was sufficient for achieving a 2-log reduction in viable counts. Repeated exposure of these two bacterial species to treatment with photolysis of H_2_O_2_ resulted in a relatively large fluctuation in the antibacterial effect compared with *S. mutans* and the four bacterial species (*S. aureus*, *E. faecalis*, *E. coli*, and *S. salivarius*) shown in Figure 3. However, even in the two species *P. aeruginosa* and *A. actinomycetemcomitans*, no development of bacterial resistance to treatment of photolysis of H_2_O_2_ was observed during 40 times of exposure. For *S. mutans*, as was the case with the former four bacterial species (*S. aureus*, *E. faecalis*, *E. coli*, and *S. salivarius*), the magnitude of reduction in viable counts hardly deviated from the range of 2- to 3-log order during repeated treatment up to 40 times.

### Quantification of hydroxyl radicals generated by photolysis of H_2_O_2_

**Figure 5:**
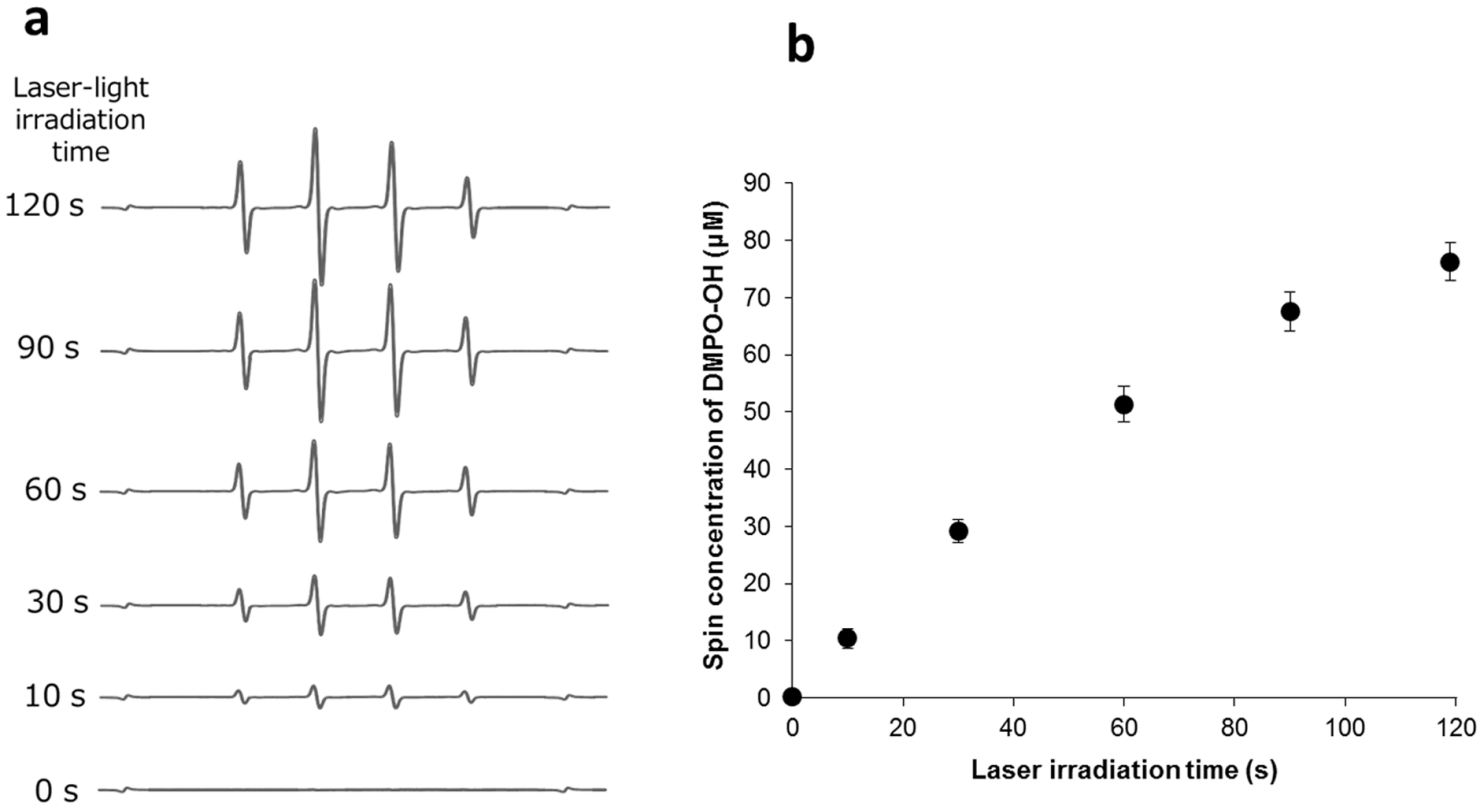
Representative ESR spectra and the yield of DMPO-OH obtained by laser-light irradiation of 3% H_2_O_2_. (a) ESR spectra and (b) DMPO-OH yields are shown. Each value in (b) represents the mean ± standard deviation (n = 3).

Laser irradiation of H_2_O_2_ generated an ESR signal of DMPO-OH. The presence of the spin adduct was confirmed by hyper fine coupling constants of a_N_ = a_H_ = 1.49 mT for DMPO-OH [17]. The yield of DMPO-OH increased linearly with the laser irradiation time, and the generation rates of DMPO-OH (slope values of lines) also increased with the concentration of H_2_O_2_ (Fig. 5). When H_2_O_2_ at concentrations of 250, 500, and 1000 mM was irradiated with the laser light for 30 s, the yields of DMPO-OH were 12.8, 22.5, and 41.6 mM, respectively.

## Discussion

The present study showed that repeated exposure of bacteria to disinfection treatment with photolysis of H_2_O_2_ did not induce bacterial resistance to this treatment. With regard to the antibacterial agents tested, in all of the agents tested, at least one of the four bacterial species resistant to the agents was observed with repeated exposure. As mentioned above, monitoring MICs of the agents after serial passage of the culture through subinhibitory concentrations of the agents has proven effective for assessing the risk of developing bacterial resistance [11]–[13]. Bacteria were cultivated under drug-free conditions prior to each susceptibility assay in the present study (Fig. 1a). The setup of the assay protocol was designed in this manner to be in accordance with susceptibility testing for disinfection treatment with photolysis of H_2_O_2_. Because continuous or serial exposure of bacteria to treatment with photolysis of H_2_O_2_ would cause a lethal effect, the serial passage technique could not be applied. Therefore, bacteria were cultivated prior to each susceptibility assay under partially bactericidal conditions, which were obtained by adjusting the laser light irradiation time (Fig. 1b). Even when cultivation was performed in advance between each susceptibility assay, repeated exposure of bacteria to subinhibitory concentrations of antibacterial agents resulted in development of bacteria that were resistant to the agents. Of the four bacterial species tested, increases in MICs were more prominent in *S. aureus* and *E. faecalis* than in *E. coli* and *S. salivarius*. The reason for the difference in the magnitude of drug-resistance induction among bacterial species cannot be explained at the present time. In addition, only one strain for each bacterial species was tested. Therefore, the conclusion that this difference was species dependent cannot be made. Nonetheless, to a greater or lesser extent, any of the bacterial species tested became resistant to one or more antibacterial agents tested. Under a similar assay protocol, disinfection treatment with photolysis of H_2_O_2_ did not result in development of resistance to this treatment in any of the four bacterial species, even after 40 exposures. With regard to the other three bacterial species, *P. aeruginosa*, *S. mutans*, and *A. actinomycetemcomitans*, disinfection treatment with photolysis of H_2_O_2_ also did not lead to development of resistance. Susceptibility of *P. aeruginosa* and *A. actinomycetemcomitans* to repeated treatment of photolysis of H_2_O_2_ fluctuated compared with the other bacterial species. In the case of *P. aeruginosa*, this was possibly due to a higher sensitivity of this bacterium than that of the other bacterial species. This could be because a laser light irradiation time as short as 10 s was sufficient to achieve a 2-log reduction in viable counts. With regard to *A. actinomycetemcomitans*, one of the possibilities for causing fluctuation might be that the bacterium was cultured under anaerobic conditions following exposure to oxidative stress by hydroxyl radicals, as well as its relatively high sensitivity to disinfection treatment. However, since such fluctuation was not observed in *S. mutans* which was also cultured under anaerobic conditions, effect of anaerobic culture conditions might not be so important.

In general, bacterial resistance is mediated through inactivation of drugs, mutation of active sites of drugs, and/or inhibition of drug-accession to active sites. In addition, bacteria resistant to more than two classes of antibiotics, which are categorized as multidrug resistant, have become a serious problem in the hospital environment. Multidrug resistance may be mediated by extra-chromosomal genetic elements or by overexpression of resistance genes in response to selective pressure [18]. In contrast to susceptibility testing for antimicrobial agents, repeated exposure of the seven bacterial species to disinfection treatment with photolysis of H_2_O_2_ did not decrease bacterial susceptibility to this treatment. This finding suggests that the risk of inducing bacterial resistance by disinfection treatment is low. In the case of photodynamic antimicrobial chemotherapy (PACT) in which exposure of a photosensitizer to light results in the formation of oxygen species (e.g., singlet oxygen and free radicals), causing microbial cell death, the development of resistance to photodynamic antimicrobial chemotherapy appears to be unlikely. This situation occurs because, in microbial cells, singlet oxygen and free radicals interact with several cell structures and different metabolic pathways [7]. The active ingredient of the disinfection treatment in the present study was the hydroxyl radical, which was laser irradiation time-dependently generated by photolysis of H_2_O_2_, but not H_2_O_2_, because exposure of bacteria to 3% (w/v) H_2_O_2_ without laser irradiation for up to 120 s did not show any bactericidal effect. In studies on PACT, Guiliani et al. studied the possible development of bacterial resistance to PACT after 20 treatments in three major human pathogens, *P. aeruginosa*, *S. aureus*, and *Candida albicans* [10]. All samples were illuminated with a fluence rate of 50 mW/cm^2^ for 10 min, and the condition allowed the pathogens survive the PACT. They demonstrated that 20 consecutive PACT treatments did not result in any resistant mutants. Similarly, Tavares et al. demonstrated that the bacteria did not develop resistance to the photodynamic process [9]. In their study, *Vibrio fischerithe* and *E. coli* were subjected to 10 repeated PACT. In their PACT with white light irradiation at 40 W/m^2^ for 25 min, 1 log unit of surviving bacteria was achieved. In our study, the disinfection treatment with photolysis of H_2_O_2_ was carried out on the second time scale. Since we have developed the disinfection treatment with photolysis of H_2_O_2_ to achieve highly effective bactericidal activity, the disinfection treatment applied in the present study has an ability to kill pathogenic bacteria including *S. aureus* and *E. faecalis* with a >5-log reduction of viable counts within 3 min [1], indicating that it is difficult to get bacteria surviving the disinfection treatment after 3 min treatment. To evaluate the risk of inducing bacterial resistance, surviving bacteria is needed to be subcultured for the next passage. That is a reason for that the treatment was carried out on the second time scale up to 120 s. To study the risk of developing bacterial resistance in this manner, not only the disinfection treatment with photolysis of H_2_O_2_ but also PACT was set to exert sublethal effect by controlling the treatment time although the disinfection treatment in the present study was carried out on the second time scale and PACT on the minute time scale. Thus despite the somewhat difference in the treatment time between the two, it is assumed that the experimental conditions were comparable each other. Therefore, as is the case with PACT, it was expected that no bacterial resistance was induced by hydroxyl radicals. Anti-oxidant enzymes, such as superoxide dismutase and catalase, protect against some reactive oxygen species, but not against hydroxyl radicals. There is the possibility that catalase in bacterial cells may affect H_2_O_2_, resulting in a reduced amount of hydroxyl radicals. However, this would be negligible because 3% H_2_O_2_ is a high enough concentration that bacteria should not be degraded by their inherent catalase. To further confirm the low risk of developing bacterial resistance, more bacterial strains including drug resistant mutants should be evaluated since only one strain of each bacterial species was tested in the present study.

Considering the emergence of antibiotic-resistant strains in recent years, disinfection treatment with photolysis of H_2_O_2_ appears to be a potential alternative for existing antimicrobial agents because of its low risk of inducing bacterial resistance.
